# Antimicrobial Medicines Consumption in Eastern Europeand Central Asia – An Updated Cross-National Study and Assessment of QuantitativeMetrics for Policy Action

**DOI:** 10.3389/fphar.2018.01156

**Published:** 2019-03-05

**Authors:** Jane Robertson, Kotoji Iwamoto, Iris Hoxha, Lilit Ghazaryan, Vafa Abilova, Ana Cvijanovic, Halina Pyshnik, Marina Darakhvelidze, Larissa Makalkina, Arianit Jakupi, Aigul Dzhakubekova, Angela Carp, Lidija Cizmovic, Svetlana Rachina, Vesela Radonjic, Salomudin Yusufi, Mesil Aksoy, Muhabbat Ibragimova, Brian Godman, Hans Kluge, Hanne Bak Pedersen

**Affiliations:** ^1^World Health Organization Regional Office for Europe, Copenhagen, Denmark; ^2^Department of Clinical Pharmacology, The University of Newcastle, Callaghan, NSW, Australia; ^3^Department of Pharmacy, University of Medicine, Tirana, Tirana, Albania; ^4^Scientific Centre of Drug and Medical Technology Expertise, Ministry of Health, Yerevan, Armenia; ^5^Department of Import Medicines and Medical Devices, Analytical Expertise Center, Ministry of Health, Baku, Azerbaijan; ^6^Sector for Providing Information on Drugs and Medical Products, Agency for Medicinal Products and Medical Devices of Bosnia and Herzegovina, Sarajevo, Bosnia and Herzegovina; ^7^Department of Pharmaceutical Inspection and Organization of Medicinal Supply, Ministry of Health of the Republic of Belarus, Minsk, Belarus; ^8^Health Care Department, Ministry of IDPs, Labour, Health and Social Affairs of Georgia, Tbilisi, Georgia; ^9^Department of Cardiology and Internal Medicine with a Course of Clinical Pharmacology and Pharmacy Astana Medical University, Astana, Kazakhstan; ^10^A2 – Pharmaceutical Consulting and UBT – Higher Education Institution, Pristina, Kosovo†; ^11^Specialized Expertise of Medicines Unit, Department of Drug Provision and Medical Devices, Ministry of Health, Bishkek, Kyrgyzstan; ^12^P.I. Coordination, Implementation and Monitoring Unit of the Health System Projects, Chişinãu, Moldova; ^13^Department for Establishing Maximum Prices and Monitoring Consumption of Medicines, Agency for Medicines and Medical Devices, Podgorica, Montenegro; ^14^Internal Medicine Department with Cardiology and Functional Diagnostics Course, Russian Friendship University, Moscow, Russia; ^15^National Centre for Information on Medicines and Medical Device, Medicines and Medical Devices Agency of Serbia, Belgrade, Serbia; ^16^Department of Pharmacy, Faculty of Medical Sciences, University of Kragujevac, Kragujevac, Serbia; ^17^Vice-Rector for Science, Avicenna Tajik State Medical University, Dushanbe, Tajikistan; ^18^Turkish Medicines and Medical Devices Agency, Department of Rational Use of Medicines, Ministry of Health, Ankara, Turkey; ^19^Head of the Information and Analytical Department, The State Center for Expertise and Standardization of Medicines, Medical Devices and Medical Equipment of the Agency for the Development of the Pharmaceutical Industry under the Ministry of Health of the Republic of Uzbekistan, Tashkent, Uzbekistan; ^20^Strathclyde Institute of Pharmacy and Biomedical Sciences, University of Strathclyde, Glasgow, United Kingdom; ^21^Liverpool Health Economics, University of Liverpool, Liverpool, United Kingdom; ^22^Division of Clinical Pharmacology, Karolinska Institute, Karolinska University Hospital Huddinge, Stockholm, Sweden; ^23^Department of Public Health Pharmacy and Management, School of Pharmacy, Sefako Makgatho Health Sciences University, Pretoria, South Africa

**Keywords:** antibiotic utilization, antimicrobial medicines consumption, national surveillance networks, cross-national comparative study, Eastern Europe, Central Asia, quality indicators

## Abstract

**Introduction:** Surveillance of antimicrobial medicines consumption is central to improving their use and reducing resistance rates. There are few published data on antibiotic consumption in Eastern Europe and Central Asia. To address this, 18 non-European Union (EU) countries and territories contribute to the WHO Regional Office for Europe (WHO Europe) Antimicrobial Medicines Consumption (AMC) Network.

**Objectives:** (i) Analyze 2015 consumption of J01 class antibacterials for systemic use from 16 AMC Network members; (ii) compare results with 2011 data and 2015 ESAC-Net estimates; (iii) assess consumption against suggested indicators; (iv) evaluate the impact of planned changes to defined daily doses (DDDs) in 2019 for some commonly used antibiotics; and (v) consider the utility of quantitative metrics of consumption for policy action.

**Methods:** Analysis methods are similar to ESAC-Net for EU countries. The Anatomical Therapeutic Chemical (ATC) classification and DDD methodology were used to calculate total consumption (DDD/1000 inhabitants/day [DID]), relative use measures (percentages), extent of use of WHO Watch and Reserve group antibiotics and impact of DDD changes.

**Findings:** Total J01 consumption in 2015 ranged 8.0–41.5 DID (mean 21.2 DID), generally lower than in 2011 (6.4–42.3 DID, mean 23.6 DID). Beta-lactam penicillins, cephalosporins, and quinolones represented 16.2–56.6, 9.4–28.8, and 7.5–24.6% of total J01 consumption, respectively. Third-generation cephalosporins comprised up to 90% of total cephalosporin consumption in some countries. Consumption of WHO Reserve antibiotics was very low; Watch antibiotics comprised 17.3–49.5% of total consumption (mean 30.9%). Variability was similar to 2015 ESAC-Net data (11.7–38.3 DID; mean 22.6 DID). DDD changes in 2019 impact both total and relative consumption estimates: total DIDs reduced on average by 12.0% (7.3–35.5 DID), mostly due to reduced total DDDs for commonly used penicillins; impact on rankings and relative use estimates were modest.

**Discussion:** Quantitative metrics of antibiotic consumption have value. Improvements over time reflect national activities, however, changes in total volumes may conceal shifts to less desirable choices. Relative use measures targeting antibiotics of concern may be more informative. Some, including WHO Watch and Reserve classifications, lend themselves to prescribing targets supported by guidelines and treatment protocols.

## Introduction

The rise in antimicrobial resistance (AMR) is a growing public health concern impacting on morbidity, mortality and costs, calling for urgent action as local problems with resistance become a global threat (Gandra et al., 2014; Michael et al., 2014; Taylor et al., 2014; O’Neill, 2015; Prestinaci et al., 2015; Jinks et al., 2016; United Nations, 2016; Jakovljevic et al., 2018). Strengthening the evidence base through surveillance of AMR and antimicrobial medicines consumption, and optimizing antimicrobial medicines use in human and animal health, are two of the five objectives of the Global Action Plan (GAP) to reduce AMR (World Health Organization, 2015). To implement the GAP, the World Health Assembly in 2015 urged Member States to develop national action plans on AMR that aligned with the objectives of the GAP (World Health Organization, 2016). Routine data collection on antimicrobial consumption in Europe predated the GAP, with reference data on antimicrobial medicines consumption from European Union (EU) member countries along with Norway and Iceland available from 1997 (European Centre for Disease Prevention and Control [ECDC], 2018). The European Surveillance of Antimicrobial Consumption (ESAC) Network (ESAC-Net) utilizes standardized methods to collect and analyze antimicrobial consumption data for both the community and the hospital sector [European Centre for Disease Control (ECDC), 2018]. Since 2011, similar methods have been used in the WHO Regional Office for Europe (WHO Europe) Antimicrobial Medicines Consumption (AMC) Network to estimate antimicrobial medicines consumption in 18 non-EU countries and territories of the WHO European region. As with ESAC-Net, data collection is based on the WHO Anatomical Therapeutic Chemical (ATC) classification system and defined daily doses (DDDs) methodology (WHO Collaborating Centre for Drug Statistics Methodology, 2018a,b). This work supplements local studies on patterns of antimicrobial consumption (Stratchounski et al., 2001; Malo et al., 2014; Smiljanic et al., 2016).

2011 data from selected countries of the WHO Europe AMC Network were published in 2014 and an analysis of AMC data for 2011–2014 published in 2017 (Versporten et al., 2014; World Health Organization Regional Office for Europe, 2017a). Both analyses reported total consumption of J01 antibacterials for systemic use (DDD per 1000 inhabitants per day [DID]), and the relative use of different pharmacological subgroups including tetracyclines, penicillins, cephalosporins, aminoglycosides, macrolides, and quinolones. These analyses also reported the relative consumption of agents recommended as second-line treatment choices including cephalosporins (particularly third- and fourth-generation agents) and quinolones on the basis that these metrics might focus attention on areas where antibiotic use could be improved. Despite some differences in data sources used and differences in levels of expenditure on medicines between Western and Eastern Europe (Jakovljevic et al., 2016), comparisons between ESAC-Net and AMC data were presented, giving a pan-European perspective on antibiotic consumption (Versporten et al., 2014; World Health Organization Regional Office for Europe, 2017a). Since then, a new classification of antibiotics introduced by WHO and some significant changes to DDD values that will take effect in 2019 have increased the range of metrics that might be reported and will affect the interpretation of some existing measures of antibiotic consumption. In addition, a number of members of the AMC Network have reviewed their antibiotic utilization patterns alongside ongoing policies to improve utilization to provide guidance for the future (Abilova et al., 2018; Bojanic et al., 2018), building on activities among European countries (Adriaenssens et al., 2011; Malo et al., 2014; Smith et al., 2014; Furst et al., 2015). There have also been activities among AMC Network countries and areas to improve the knowledge and activities of community pharmacists regarding the treatment of infections in ambulatory care as pharmacists are often the first point of contact for patients (Markovic-Pekovic et al., 2017; Hoxha et al., 2018). This builds on proposed WHO activities among pharmacists in Europe (World Health Organization Regional Office for Europe, 2014).

In April 2017, the Expert Committee on the Selection and Use of Essential Medicines recommended changes to the WHO Model Lists of Essential Medicines for adults (EML) and children (EMLc), following a comprehensive review of sections 6.2.1 (Beta-lactam medicines) and 6.2.2 (Other antibacterials) (World Health Organization, 2017c). After reviewing up-to-date evidence summaries on the treatment of 21 priority infectious conditions (based on disease burden, severity of illness and prospects for improving antibiotic use), five pediatric infectious syndromes and several sexually transmitted infections (World Health Organization, 2017a), the Committee identified empirical first- and second-choice treatments for common, community-acquired infections, focusing on treatment choices broadly applicable in most countries (Sharland et al., 2018). The Expert Committee also proposed a categorization of antibiotics into Access, Watch, and Reserve groups (Boxes 1, 2), taking account of recommendations of WHO and OIE (World Organisation for Animal Health) for the management of antibacterials that are critically important for both human and animal health (World Organisation for Animal Health, 2015; World Health Organization, 2017b). Not all medicines on the Model Lists were assigned to the three groups, leaving a fourth “ungrouped” category, with the classification to be revised as additional clinical syndromes are reviewed (Sharland et al., 2018). However, this classification could support antimicrobial stewardship efforts and focus attention on prescribing practices that should be further reviewed.

**Box 1:** WHO categories of antibiotics – descriptions.

Group	Definition
Access group	First- and second-choice antibiotics that should be widely available in all countries. They should be affordable and quality assured.
Watch group	First- and second-choice antibiotics that only should be used for a specific, limited number of indications due to higher resistance potential.
Reserve group	Last resort antibiotics that should be used only when other antibiotics have failed or for infections of multi-resistant bacteria.


**Box 2:** Medicines assigned to WHO access, watch, and reserve groups.

Access group			

Medicine	ATC code#	Medicine	ATC code#
Beta-lactam medicines		Other antibacterials	
Amoxicillin	J01CA04	Amikacin	J01GB06
Amoxicillin + clavulanic acid	J01CR02	Azithromycin^∗^	
Ampicillin	J01CA01	Chloramphenicol	J01BA01
Benzathine benzylpenicillin	J01CE08	Ciprofloxacin^∗^	
Benzylpenicillin	J01CE01	Clarithromycin^∗^	
Cefalexin	J01DB01	Clindamycin	J01FF01
Cefazolin	J01DB04	Doxycycline	J01AA02
Cefixime^∗^		Gentamicin	J01GB03
Cefotaxime^∗^		Metronidazole	J01XD01, P01AB01
Ceftriaxone^∗^		Nitrofurantoin	J01XE01
Cloxacillin	J01CF02	Spectinomycin	J01XX04
Phenoxymethylpenicillin	J01CE02	Sulfamethoxazole + trimethoprim	J01EE01
Piperacillin + tazobactam^∗^		Vancomycin (oral and parenteral)^∗^	
Procaine benzylpenicillin	J01CE09		

**Watch group**	

**Medicine**			**ATC code**

Quinolones and fluoroquinolones e.g., ciprofloxacin, levofloxacin, moxifloxacin, norfloxacin			J01MA, J01MB
Third-generation cephalosporins (with or without beta-lactamase inhibitor) e.g., cefixime, ceftriaxone, cefotaxime, ceftazidime			J01DD
Macrolides e.g., azithromycin, clarithromycin, erythromycin			J01FA
Glycopeptides e.g., teicoplanin, vancomycin			J01XA, A07AA09
Antipseudomonal penicillins with beta-lactamase inhibitor e.g., piperacillin + tazobactam			J01CR03, J01CR05
Carbapenems e.g., meropenem, imipenem + cilastatin			J01DH
Penems e.g., faropenem			J01DI03

**Reserve group**	

**Medicine**			**ATC code**

Aztreonam			J01DF01
Fourth-generation cephalosporins e.g., cefepime			J01DE
Fifth-generation cephalosporins e.g., ceftaroline			J01DI02, J01DI01, J01DI54
Fosfomycin IV			J01XX01 (Only parenteral)
Oxazolidinone e.g., linezolid			J01XX08, J01XX11
Polymyxins e.g., polymyxin B, colistin			J01XB, A07AA10, A07AA05
Tigecycline			J01AA12
Daptomycin			J01XX09


*^∗^Watch group antibiotics included in the EML/EMLc only for specific, limited indications; #ATC codes shown for medicines in the core access list – i.e., no overlap with Watch group antibiotics.*

*Medicines not specifically identified in the groups described form an “ungrouped” medicines category.*

Following an application from the European Centre for Disease Control (ECDC), in October 2017 the WHO International Working Group for Drug Statistics Methodology recommended changes to the DDDs for seven commonly used antibiotics (mainly penicillins), and endorsed new DDDs for oral colistin along with changes for a number of other products (Box 3) (WHO Collaborating Centre for Drug Statistics Methodology, 2018c). The changes were requested given evidence that current DDD allocations for commonly used medicines differed substantially from recommended doses and doses used in clinical practice. The DDD changes will be fully adopted in 2019 and will affect estimates of total antibiotic medicines consumption, relative use of classes of antibiotics and interpretation of national and cross-national comparisons over time.

**Box 3:** 2019 changes to DDDs for commonly prescribed J01 antibacterials.

ATC code	Medicine	Current DDD	New DDD
J01CA04	Amoxicillin	1 g O	1.5g O
J01CA04	Amoxicillin	1 g P	3g P
J01CR02	Amoxicillin and beta-	1 g O	1.5g O
	lactamase inhibitor
J01CA01	Ampicillin	2 g P	6g P
J01DE01	Cefepime	2 g P	4g P
J01DH02	Meropenem	2 g P	3g P
J01MA02	Ciprofloxacin	0.5 g P	0.8g P
J01XB01	Colistin	3 MU P	9 MU P


**Source: https://www.whocc.no/atc/lists_of_new_atc_ddds_and_altera/alterations_in_atc_ddd/. ATC, Anatomical Therapeutic Chemical; DDD, Defined Daily Dose; g, gram; O, oral; P, parenteral; MU, million unit. Other changes to DDDs that will apply in 2019: New DDDs are assigned for kanamycin oral (A07AA08); colistin oral (A07AA10); cefroxadine (J01DB11); cefteram (J01DD18); ceftriaxone and beta-lactamase inhibitor (J01DD63); tebipenem pivoxil (J01DH06); faropenem (J01DI03); midecamycin (J01FA03); lomefloxacin (J01MA07); gemifloxacin (J01MA15): garenoxacin (J01MA19); tosufloxacin (J01MA22); and delafloxacin (J01MA23).**

This study presents updated data from the WHO Europe AMC Network using cross-national comparisons of 2015 antibiotic consumption data for 16 network members where the Ministry of Health approved data sharing and publication. Consumption estimates for 2011 and 2015 are compared; the WHO “Watch” and “Reserve” classification of antibiotics is applied, and the impact of proposed changes to DDDs in 2019 are examined. In addition, we consider the information value of different quantitative metrics to policymakers, consumers, and health care professionals to provide future guidance.

## Materials and Methods

### Participating Countries and Areas

Sharing of the 2015 data was approved for 16 of 18 AMC Network members – Albania, Armenia, Azerbaijan, Bosnia and Herzegovina, Belarus, Georgia, Kazakhstan, Kyrgyzstan, Republic of Moldova, Montenegro, Russian Federation, Serbia, Tajikistan, Turkey, Uzbekistan, and Kosovo [in accordance with Security Council resolution 1244 (1999)].

### Data Collection

The methods used in the AMC Network have been described elsewhere (Versporten et al., 2014; World Health Organization Regional Office for Europe, 2017a). Briefly, data collection follows a standardized protocol using an Excel template based on a national register of antimicrobial medicines with marketing authorization. Products are identified by ATC code facilitating analyses from the main class (level 1) to individual medicines (level 5). National AMC focal points enter data on the numbers of packages of each product imported or sold in their country, relevant product information, population data, and assigned DDD (World Health Organization Regional Office for Europe, 2017a; Table A1).

The core medicines monitored are the antibacterials for systemic use (ATC group J01); antibiotics for alimentary tract and metabolism (A07AA); and nitroimidazole derivatives against amoebiasis and other protozoal diseases (P01AB). There is optional data collection for antimycotics (J02), antifungals (D01BA), and antivirals for systemic use (J05), drugs for treatment of tuberculosis (J04A) and antimalarials (P01B). Only data for the core medicines are presented here.

### Data Sources

Import data from customs records and declaration forms is the most commonly reported source of information supplemented with sales records from market authorization holders, local manufacturing estimates, wholesaler records, and in some cases, commercial data sources (see Supplementary Table 1). Data represent total consumption estimates with the exception of Kazakhstan where a commercial data source provides coverage of around 80–85% of hospital and community sales.

### Data Validation

A template macro detects missing compulsory data and incorrect data units. WHO Europe also reviews entries to identify data inconsistencies, improbable estimates, errors in estimates for combination products when converted to standardized units, and a final data set for analysis is agreed.

### Data Analysis and Metrics Reported

Total numbers of DDDs for each product are aggregated to give the total number of DDDs at the desired ATC code level and adjusted for population to calculate DID. World Bank population estimates were applied apart from Turkey, where estimates were adjusted to take account of the large refugee and displaced persons populations. Results are compared to publicly available 2015 ESAC-Net data that are derived using similar methods (European Centre for Disease Control [ECDC], 2017).

Measures of relative consumption, expressed as a percentage of total consumption of groups of antimicrobials, were derived for pharmacological subgroups of J01, cephalosporins and quinolones as a proportion of total J01 consumption, cephalosporins by generation of agent, and for WHO Watch and Reserve groups of antibiotics. These relative use measures demonstrate the extent of consumption of second-line and last resort antibiotics. Means, medians, and ranges of estimates are presented to illustrate the variability of the 16 national datasets.

Changes in total and relative consumption between 2011 and 2015 are reported. The impact of changes to assigned DDDs in 2019 is explored for total consumption and relative consumption estimates by applying DDDs for 2019 to numbers of packages consumed in 2015. Change was calculated as the percentage increase or decrease in total DDDs from the 2015 estimate using 2016 DDD values.

### Role of the Funding Source

The funder (Netherlands Ministry of Health, Welfare and Sport) had no role in study design, data collection, data analysis, data interpretation, or writing the report.

## Results

**FIGURE 1:**
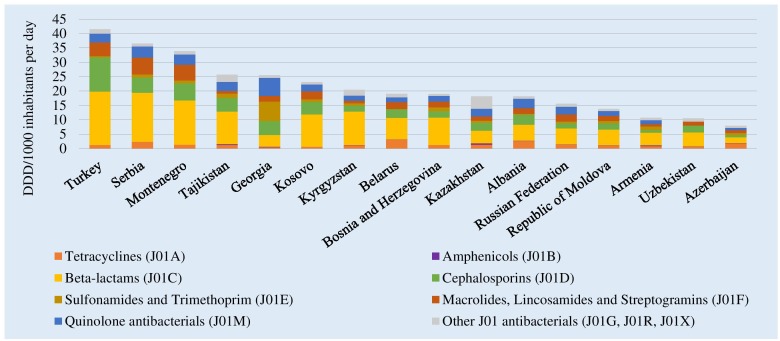
Total consumption of J01 antibacterials by pharmacological subgroup in 15 countries and Kosovo, 2015. DDD, Defined Daily Dose; ^∗^Kosovo [in accordance with United Nations Security Council resolution 1244 (1999)]; #Kazakhstan: commercial data source provides coverage of around 80–85% of hospital and community sales.

Total antibiotic consumption (ATC group J01) in 2015 ranged from 41.5 DID in Turkey to 8.0 DID in Azerbaijan (Figure 1). Mean and median consumption were 21.2 and 19.0 DID, respectively. The relative use of parenteral formulations varied from 4% in Turkey and Bosnia and Herzegovina to 52% in Uzbekistan (see Supplementary Figure 1). Beta-lactam penicillins (ATC group J01C) were the most commonly used antibiotics in almost all AMC Network countries, ranging from 56.6% of total J01 consumption in Kyrgyzstan to 16.2% in Georgia. In Georgia, highest relative consumption was reported for medicines in the sulfonamides and trimethoprim group (J01E, 26.4% of total consumption).

ESAC-Net estimates from 25 countries reporting both community and hospital sector data in 2015 show similar variability with a range from 38.3 DID in Greece to 11.7 DID in Netherlands, with mean and median consumption of 22.6 and 22.2 DID, respectively [European Centre for Disease Control (ECDC), 2017].

**FIGURE 2:**
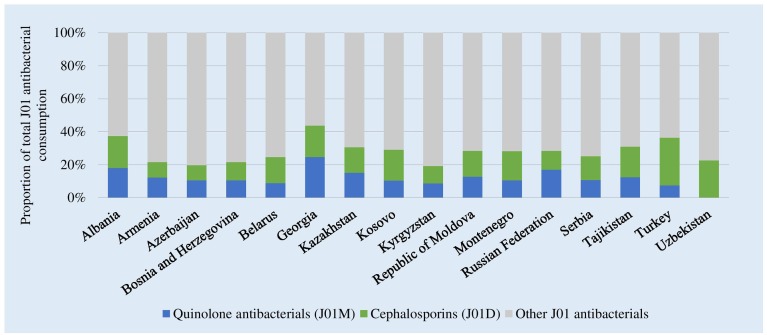
Consumption of cephalosporins and quinolones as a proportion of the total consumption of J01 antibacterials, 2015. ^∗^Kosovo [in accordance with United Nations Security Council resolution 1244 (1999)].

Figure 2 shows the consumption of cephalosporins and quinolones as a proportion of total consumption of J01 antibacterials. Highest relative consumption of cephalosporins was reported in Turkey (28.8%,) and lowest in Azerbaijan (9.2%). Highest relative quinolone consumption was in Georgia (24.6%). Together these two groups combined represented 44% of total J01 consumption in Georgia to 20% of consumption in Kyrgyzstan.

**FIGURE 3:**
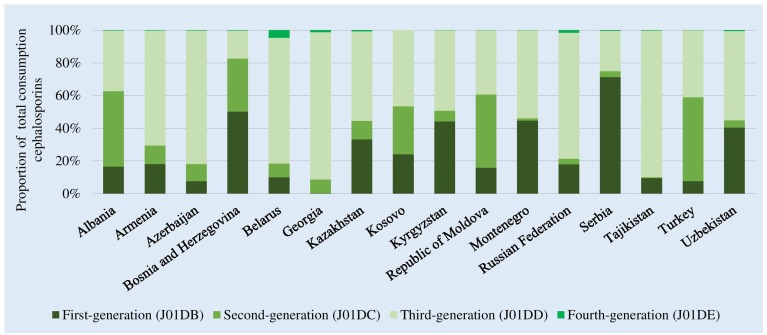
Relative consumption of cephalosporins by generation of agents, 2015. ^∗^Kosovo [in accordance with United Nations Security Council resolution 1244 (1999)].

There were high levels of consumption of first- and second-generation cephalosporins in Bosnia and Herzegovina (82% of total cephalosporin consumption) and Serbia (75%). Consumption of third-generation agents that are mostly second-line treatment options, and included in the WHO Watch list, dominated in almost half of the participating countries and represented up to 90% of total cephalosporin consumption in Tajikistan and Georgia (Figure 3). Reported consumption of fourth-generation agents (Reserve category) was low.

### WHO Watch and Reserve Category Medicines

**FIGURE 4:**
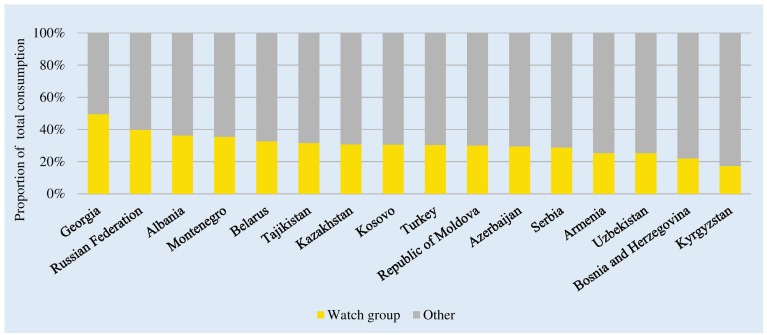
Consumption of “Watch” group of antibiotics classes as a proportion of total consumption of antibacterials. Watch group: Quinolones (J01MA and J01MB); 3^rd^ generation cephalosporins (J01DD); macrolides (J01FA); glycopeptides (J01XA and A07AA09); antipseudomonal penicillins with beta-lactamase inhibitor (J01CR03 and J01CR05); carbapenems (J01DH); and faropenem (J01DI03). ^∗^Kosovo [in accordance with United Nations Security Council resolution 1244 (1999)].

There were no or very low levels of consumption of Reserve group antibiotics across the studied countries (0.01–0.83% of total consumption; see Supplementary Table 2). Watch group antibiotics comprised 49.5% of total J01 consumption in Georgia to 17.3% of consumption in Kyrgyzstan (Figure 4). The mean and median relative consumption of Watch group antibiotics were 30.9 and 30.5%, respectively.

### Changes Over Time

There were decreases in total consumption estimates between 2011 and 2015 in nine countries, increases in five and in one remaining reasonably stable over time (Table 1). Mean J01 consumption decreased from 23.6 to 21.2 DID between 2011 and 2015, and median consumption from 22.0 to 19.0 DID.

**Table 1:** Total antibiotic consumption 2011, 2015 in DDD/1000 inhabitants per day.

	J01 antibiotic consumption DDD/1000 inhabitants per day	
	
	2011	2015
Albania	25.1	18.2
Armenia	15.9	10.7
Azerbaijan	17.1	8.0
Bosnia and Herzegovina	18.4	19.0
Belarus	17.9	19.0
Georgia	22.0	25.5
Kazakhstan#	-	18.2
Kyrgyzstan	24.0	20.5
Republic of Moldova	21.3	13.8
Montenegro	38.3	33.9
Russian Federation	15.3	15.5
Serbia	26.4	36.5
Tajikistan	36.6	25.7
Turkey	42.3	41.5
Uzbekistan	6.4	10.5
Kosovo^∗^	26.4	23.1


*#commercial data source provides coverage of around 80–85% of hospital and community sales; ^∗^in accordance with United Nations Security Council resolution 1244 (1999).*

Within these changes in total DIDs, there were some substantial changes in the reported consumption of parenteral formulations: decreasing relative consumption in Azerbaijan (47–22%) and Kyrgyzstan (72–42%) and increasing in Georgia (16–36%) and Uzbekistan (42–52%).

Between 2011 and 2015, there were substantial increases in relative consumption of the cephalosporin and quinolones combined in Albania (22–37%), Azerbaijan (8–20%), Georgia (19–44%), and Kyrgyzstan (9–20%). Conversely, there were reductions in the consumption of these groups combined in Turkey (42–37%) and Uzbekistan (33–23%).

### Changes to DDDs in 2019

Applying the DDD changes to be implemented in 2019 to consumption data for 2015 reduced total consumption estimates from a range of 41.5–8.0 DID using 2016 DDD values to 35.5–7.3 DID using 2019 DDD values (Table 2). The percentage reductions in total DIDs ranged from 18.4 (Uzbekistan) to 4.3% (Kazakhstan), with mean and median DID reductions of 12.0 and 13.0%, respectively, mostly due to reduced total DDDs for penicillins. However, there was limited impact on rankings from highest to lowest total consumption in DIDs (Table 2).

**Table 2:** Impact of 2019 changes in DDDs on estimates of total antibiotic consumption and ranking of countries.

	2016 DDD values		2019 DDD values			
		
	Rank^∗^	DID#	Rank^∗^	DID#	% decrease	Change in rank
Turkey	1	41.5	1	35.5	14.5	–
Serbia	2	36.5	2	31.0	15.1	–
Montenegro	3	33.9	3	29.0	14.4	–
Tajikistan	4	25.7	5	21.6	16.0	-1
Georgia	5	25.5	4	24.2	5.1	+1
Kosovo§	6	23.1	6	20.0	13.6	–
Kyrgyzstan	7	20.5	8	16.7	18.4	-1
Belarus	8	19.0	9	17.0	10.3	-1
Bosnia and Herzegovina	9	19.0	11	16.2	14.7	-2
Kazakhstan	10	18.2	7	17.4	4.3	+3
Albania	11	18.2	10	16.3	10.5	+1
Russian federation	12	15.5	12	14.1	9.2	
Republic of Moldova	13	13.8	13	12.9	7.0	–
Armenia	14	10.7	14	9.4	12.5	–
Uzbekistan	15	10.5	15	8.6	18.4	–
Azerbaijan	16	8.0	16	7.3	7.9	–
Mean		21.0		18.4		
Median		19.0		16.5		
Range		8.0–41.5		7.3–35.5		


*^∗^highest to lowest; #DID DDD/1000 inhabitants per day; §in accordance with United Nations Security Council resolution 1244 (1999).*

While absolute consumption estimates for cephalosporins and quinolones changed very little, relative consumption of the two groups combined increased slightly from 1.3% in Kazakhstan to 6.1% in Tajikistan (data not shown). Changes to DDD values increased the estimate of relative consumption of Watch group antibiotics – 17–49% of total consumption using 2016 DDD values and 21–52% using 2019 DDD values (data not shown).

## Discussion

Our main findings are that total antibiotic consumption in 2015 ranged from 8.0 DID for Azerbaijan to 41.5 DID for Turkey (mean 21.2 DID). These estimates were mostly lower than those reported in 2011 (range 6.4 DID Uzbekistan to Turkey 42.3 DID, mean 23.6 DID). There were increases in relative consumption of parenteral formulations, cephalosporins (particularly third-generation agents), and quinolones that are included in the WHO Watch list of antibiotics in several countries. Changes to DDDs to be implemented in 2019 impacted on both total and relative consumption estimates, driven mostly by DDD changes for several commonly used beta-lactam penicillins. The impact of the DDD changes on ranking of countries by total consumption estimates was modest. Where the relative consumption of beta-lactams was similar, there were similar percentage reductions in total DIDs.

These observations are important given the paucity of published data on antibiotic consumption from AMC network countries and areas. An IQVIA MIDAS-based study used national sample surveys of 2015 antibiotic sales extrapolated to national sales volumes for 76 countries and reported quantitative estimates of consumption for only two AMC Network members – Turkey and Russian Federation (Klein et al., 2018). We report total consumption of 41.5 DID for Turkey compared to Klein’s estimate of around 47 DID. There are likely several reasons for these differences including the extrapolation and interpolation algorithms applied to IQVIA data to generate national estimates of consumption and the assignment of DDDs for all products without a formal WHO DDD value. Medicines without an assigned DDD are excluded from our analyses. Klein et al. (2018) demonstrated good correlation of their estimates with ESAC-Net data; however, it may reflect that IQVIA data collection in EU countries is more comprehensive than in other parts of the world, reducing the impact of the extrapolations applied. The difference in absolute estimates of consumption in Turkey raises questions about using such data for “setting and enforcing per capita consumption targets” at the national level (Klein et al., 2018). AMC Network data for Russian Federation are based on IQVIA data, perhaps explaining the much closer estimates of 15.5 and 16 DIDs from the two sources. Further work is needed to compare consumption estimates from different data sources – import records, wholesaler data, health insurance records, and other commercial information sources – as the need is for reliable, actionable data on antibiotic consumption that can be used to monitor the impact of interventions to change prescribing practices. We are aware for instance that there can be considerable variation with import data from month to month.

Our estimates have some limitations, relying on a complete register of antimicrobial products, full and accurate reporting of data, distinguishing between medicines for local consumption and export, and the impact of import cycles. In the absence of universal health coverage or e-prescribing, widespread availability of antibiotics without prescription, few mechanisms to engage private wholesalers and limited ability to disaggregate data to hospital and community sectors, import records remain the most feasible data source in most AMC Network countries and areas. However, the broad comparability of total consumption estimates for AMC Network and ESAC-Net countries suggest that estimates reported here are plausible and can provide a platform for national level discussions and actions.

Reported decreases in total DIDs in this study are credible, reflecting sustained efforts at the national level to improve antibiotics use. Turkish health authorities have implemented an electronic prescription system to track prescription data and provide feedback to physicians and adopted a Rational Drug Use National Action Plan 2014–2017 that prioritizes the appropriate use of antibiotics (World Health Organization Regional Office for Europe, 2017b). 2011 Turkey estimates (42.3 DID) relied on commercial IMS data and reflected community consumption; the comparable community estimate in 2015 was 40.0 DID, derived from the comprehensive pharmaceutical “track and trace” system that follows medicines from production to consumption (Government of Turkey, 2018). 2015 data are also unlikely to reflect the full impact of the sustained educational and regulatory interventions undertaken since 2014.

The reduction in total antibiotic consumption from 15.9 to 10.7 DID between 2011 and 2015 in Armenia is likely related to targeted awareness activities conducted by the multisectoral team of the Ministry of Health. Multiple ongoing reforms and activities in Azerbaijan targeting physicians, pharmacists and patients are suggested to have contributed to reducing total antibiotic consumption from 17.1 to 8.0 DID in the same time period (Abilova et al., 2018). However, changes in pricing policies have seen a shift from penicillins toward consumption of tetracyclines, and while overall consumption of cephalosporins was low, more than 80% was third-generation agents. Pricing policies aimed at improving affordability and access has affected market dynamics, with some manufacturers leaving the market, influencing medicine choices (Abilova et al., 2018). Pricing policies could be used to drive more appropriate use, for example, lower prices for first- and second-generation cephalosporins than third-generation agents (Abilova et al., 2018).

More modest increases in total consumption in Georgia (22.0 DID in 2011 to 25.5 DID in 2015) may reflect, in part, issues in data collection, as exported products are not fully accounted for. Nonetheless, there appear to be some substantial shifts in consumption patterns, with reductions in relative consumption of beta-lactams (67.7–16.2%), increases in cephalosporins (8.8–19.2%) with more than 90% third-generation agents, and increases in quinolone consumption (10.5–24.6% of total J01 consumption). These observations highlight the importance of metrics other than total volumes of use. The choice of antimicrobial is as important as the volume of use given increasing concerns with AMR, and may be a more amenable target for changes in prescribing practices and optimizing antibiotic use when supported by evidence-based guidelines (World Health Organization, 2017a; Klein et al., 2018). Serbia reported increased consumption from 26.4 to 36.5 DID between 2011 and 2015. The reasons for this require local level investigation. However, limited resources have compromised the implementation of physician education programs, promotion of rational use of antibiotics and hospital-based antimicrobial stewardship programs (Kalaba et al., 2018).

The DRIVE-AB project distinguishes between prescribing quality indicators and quantitative metrics, proposing 51 inpatient and 32 outpatient quality indicators covering aspects including stewardship, diagnostics, dosing, duration, safety and monitoring of antibiotics, as well as 12 inpatient and 6 outpatient quantity metrics (DRIVE-AB, 2014). A *quality indicator* reflects the degree in which the antibiotic is correct or appropriate, where the outcome has a value on its own. A *quantity metric* reflects the volume or costs of antibiotic use and the outcome only gains value in its comparison. By these definitions, most of the metrics presented here are quantitative metrics, although quantitative measures focusing on preferred agents might be considered pointing toward improved prescribing practices and some measure of quality. This is the first step though to improve future antibiotic use in the absence of patient level data, especially given the high rate of self-purchasing of antibiotics without a prescription in a number of network countries.

Metrics such as packages of medicines per 1000 inhabitants per day have been proposed as an alternative to DIDs in the outpatient setting, partly in response to differences in prescribed daily doses in different countries (Bruyndonckx et al., 2014; Coenen et al., 2014; Watier et al., 2017). However, consumption estimates are affected by choice of measurement unit underpinning the importance of the use of the same data sources and metrics over time for assessment of temporal trends and benchmarking (Watier et al., 2017). Patient-linked volume of use measures are being used as national prescribing targets, with Sweden adopting a long-term goal of 250 prescriptions per 1000 inhabitants/year for all age groups, and the 2016 UK government proposing to halve inappropriate prescribing by 2020 (Government of Sweden, 2016; UK Department of Health Media Centre, 2016). However, prescription data are not available in all settings especially among AMC member countries and areas. Assessment of appropriate use (quality indicators) requires patient-level information linking clinical condition, patient characteristics and prescribing choices. Clinicians will more likely respond to these data than higher level aggregate measures. As health information systems develop, it will become possible to move beyond quantitative metrics toward quality indicators. In the interim, focused studies such as point prevalence studies, prescription analyses, and community surveys supplemented with qualitative studies are being undertaken in AMC Network countries and areas to help fill the information gaps and provide evidence of practices that should be reviewed (Smiljanic et al., 2016). There are also ongoing activities to improve pharmacist and patient knowledge to reduce inappropriate dispensing of antibiotics especially for upper respiratory tract infections (Markovic-Pekovic et al., 2017; Hoxha et al., 2018).

Policymakers and consumers require simple metrics that are easily interpretable, identify the magnitude of problems with antibiotic consumption and suggest the need for policy actions such as regulations, the enforcement of prescription-only status and investments in education and training. In the absence of prescription data, total consumption in DID could be used for this purpose although is difficult to interpret in isolation requiring trend data at the national or cross-national level to provide some context (Van Boeckel et al., 2014; Versporten et al., 2014; World Health Organization Regional Office for Europe, 2017a). DDD changes in 2019 will likely compound the problems of interpretation of consumption estimates, with total DIDs decreasing on average by around 12% with the new DDDs applied, independent of any intervention by government, agencies or professional groups. Communication strategies will be required so stakeholders are aware of the impact of the DDD changes along with re-setting of trend lines and targets for changes in antibiotic consumption at the national level.

The WHO Watch and Reserve group classifications offer promise as metrics that indicate actions required and lend themselves to prescribing targets with lower absolute and relative levels of consumption of these groups of antibiotics desirable. The Access, Watch, and Reserve classifications are already being applied – to IQVIA sales data units for single molecule and combination antibiotic products in India, and in the Access to Medicines Foundation analysis of proportions of pharmaceutical companies’ marketed antibiotics that are listed in the WHO EML antibiotic groups (McGettigan et al., 2017; Access to Medicines Foundation, 2018). More relevant for national stewardship efforts is an analysis of relative consumption of antibiotics by the WHO groups. However, a standardized method of calculation (ATC codes included and denominator definitions) is needed to ensure the validity of comparisons between settings (community, hospital, and total consumption) and for monitoring changes over time. The estimates presented here are based on total consumption – the relative use of Watch and Reserve groups would be substantially higher in a hospital-based analysis. The lists of Watch and Reserve medicines will be modified as evidence emerges, and more clinical conditions are reviewed.

While the quantitative metrics presented have limited application in assessing the appropriateness of prescribing, they do illustrate differing patterns of antibiotic consumption between countries and within countries over time and point to potential problems in antibiotic use. A full exploration of reasons for the changes in consumption patterns reported in each of the 16 AMC Network countries and areas is beyond the scope of this study. However, the impact of locally produced antibiotics on treatment choices, pharmaceutical industry promotion, perverse incentives to prescribe and dispense antibiotics, availability and use of up-to-date guidelines all need to be considered in developing interventions to improve antibiotic use. Quantitative measures can be effective. Turkish health authorities responded to high levels of antibiotic consumption with substantial commitments of resources and integrated activities to improve antibiotic use (World Health Organization Regional Office for Europe, 2017b). Relative use measures targeting less appropriate treatment choices (third- and fourth-generation cephalosporins, quinolones, Watch and Reserve antibiotics) may be more effective with clinicians and provide targets for action supported by evidence-based guidelines and treatment protocols. Other measures such as the removal of incentives to prescribe and dispense antibiotics, enforcement of prescription-only status, better diagnostics, favoring narrow-spectrum over broad-spectrum antibiotics and raising awareness and education of the public regarding the importance of preserving the value of existing antibiotics are also required. These are considerations for the future among AMC Network countries and areas, and we will be reporting further on this in the future.

## Data Availability

The datasets used in these analyses are available upon request to interested researchers.

## Ethics Statement

Ethics committee approval is not required for this study that is based on aggregate data from customs records and declaration forms, sales records from market authorization holders, local manufacturing estimates, wholesaler records, and in some cases, commercial data sources. We have obtained signed declarations from the Ministries of Health of the 15 countries and one territory included in this study giving permission for data sharing and publication.

## Author Contributions

JR, KI, and HBP developed the concept. IH, LG, VA, ACv, HP, MD, LM, AJ, AD, ACa, LC, SR, VR, SY, MA, and MI (national focal points) collected and validated national data. JR and KI undertook analysis and interpretation of data in consultation with the national focal points. JR provided first write-up of the manuscript. All co-authors KI, HBP, BG, HK, IH, LG, VA, ACv, HP, MD, LM, AJ, AD, ACa, LC, SR, VR, SY, MA, and MI reviewed and critiqued the manuscript and agreed to submission of the manuscript.

## Disclaimer

The authors alone are responsible for the views expressed in this publication and they do not necessarily represent the views, decisions or policies of the institutions with which they are affiliated.

## Conflict of Interest Statement

The authors declare that the research was conducted in the absence of any commercial or financial relationships that could be construed as a potential conflict of interest. The reviewer MJ declared a shared affiliation, with no collaboration, with one of the authors, VR, to the handling Editor at the time of review.

## APPENDIX

**Table A1:** List of AMC focal points and contributors to the 2015 data collection.

Albania	Iris Hoxha	Department of Pharmacy, University of Medicine, Tirana
Armenia	Lilit Ghazaryan^∗^	Scientific Centre of Drug and Medical Technology Expertise of Ministry of Health
Azerbaijan	Vafa Abilova^∗^	Department of Import Medicines and Medical Devices, Analytical Expertise Center, Ministry of Health
	Nazifa Mursalova^∗^	Sector of Sanitary Epidemiological Surveillance Ministry of Health
Bosnia and Herzegovina	Ana Cvijanovic	Sector for Providing Information on Drugs and Medical Products in Agency for Medicinal Products and Medical Devices of Bosnia and Herzegovina
	Tijana Spasojevic^∗^	Sector for Providing Information on Drugs and Medical Products in Agency for Medicinal Products and Medical Devices of Bosnia and Herzegovina
Belarus	Halina Pyshnik^∗^	Department of Pharmaceutical Inspection and Organization of Medicines Supply, Ministry of Health
Georgia	Marina Darakhvelidze^∗^	Health Care Department, Ministry of IDPs, Labour, Health and Social Affairs of Georgia
	Marine Baidauri	Regulations Division of Health Care Department, Ministry of IDPs, Labour, Health and Social Affairs of Georgia
	David Tsereteli	National Center for Disease Control and Public Health
Kazakhstan	Larissa Makalkina	Department of Cardiology and Internal Medicine with a course of clinical pharmacology and Pharmacy, Astana Medical University
	Zhannat Asina	Kazan Federal University, Alphateam LLP
Kyrgyzstan	Ismailova Baktygul Abdyldaevna^∗^	Public Health Unit, Ministry of Health
	Aigul Dzhakubekova^∗^	Unit on Specialized Expertise of Medicines of Department of Drug Provision and Medical Devices, Ministry of Health
Republic of Moldova	Nicolae Furtuna^∗^	National Public Health Centre
	Angela Carp	P.I. Coordination, Implementation and Monitoring Unit of the Health System Projects
Montenegro	Lidija Cizmovic^∗^	Department for Establishing Maximum Prices and Monitoring Consumption of Medicines, Agency for Medicines and Medical Devices
Russian Federation	Svetlana Rachina	Internal Medicine Department with Cardiology and Functional Diagnostics Course named after academician V.S. Moiseev, Russian Friendship University, Moscow, Russian Federation, Interregional Association for Clinical Microbiology & Antimicrobial Chemotherapy, Russian Federation
Serbia	Vesela Radonjic^∗^	National Centre for Information on Medicines and Medical Device, Medicines and Medical Devices Agency of Serbia
		Department of Pharmacy, Faculty of Medical Sciences, University of Kragujevac, Serbia
Tajikistan	Nargis Maqsudova	Health Systems, WHO Country Office in Tajikistan
	Salomudin Yusufi	Vice-Rector for Science, Avicenna Tajik State Medical University
Turkey	Melda Kecik	Department of Rational Use of Medicines. Turkish Medicines and Medical Devices Agency, Ministry of Health of Turkey
	Mesil Aksoy^∗^	
	Ali Alkan	Vice Presidency of Medicines and Pharmacy Turkish Medicines and Medical Devices Agency, Ministry of Health of Turkey
	Hakki Gursoz	Turkish Medicines and Medical Devices Agency, Ministry of Health of Turkey,
	Bahar Melik	
	Fatma İsli^∗^	
	Emre Umut Gurpinar	
	Serap Tekbacak	
	Omer Hakan Simsek	
Uzbekistan	Muhabbat Ibragimova^∗^	The State Center for Expertise and Standardization of Medicines, Medical Devices and Medical Equipment of the Agency for the Development of the Pharmaceutical Industry under the Ministry of Health of the Republic of Uzbekistan
Kosovo^#^	Arianit Jakupi^∗^	A2 - Pharmaceutical Consulting and UBT - Higher Education Institution

*^∗^Nominated AMC focal points; ^#^in accordance with Security Council resolution 1244 (1999).*
