# Supplementary material for: Antimicrobial Medicines Consumption in Eastern Europeand Central Asia – An Updated Cross-National Study and Assessment of QuantitativeMetrics for Policy Action
Source: Front Pharmacol. 2019 Mar 5;9:1156. doi: 10.3389/fphar.2018.01156 (PMC6411709; doi:10.3389/fphar.2018.01156)
Supplement: Supplementary file 1 [file Table_1.docx]

**ROBERTSON_SUPPLEMENTARY TABLES**

**Table 1 Sources of data used for consumption estimates (2011 and 2015)**

| Country or area | Years of data | Health care sector coverage | Data sources for consumption estimates |
| --- | --- | --- | --- |
| Albania | 2011, 2015 | Total care | Import records |
| Armenia | 2011, 2015 | Total care | Import records  Sales records from local manufacturers |
| Azerbaijan | 2011, 2015 | Total care | Import records |
| Bosnia and Herzegovina | 2011, 2015 | Total care | Sales records of wholesalers and local manufacturers |
| Belarus | 2011, 2015 | Total care | Import records  Sales records from local manufacturers |
| Georgia | 2011, 2015 | Total care | Import records |
| Kazakhstan | 2015 | Total care# | VIORTIS |
| Kyrgyzstan | 2011, 2015 | Total care | Import records  Sales records from wholesalers |
| Montenegro | 2011, 2015 | Total care | Sales records from wholesalers |
| Republic of Moldova | 2011, 2015 | Total care | Import records  Manufacturing records from local manufacturers |
| Russian Federation | 2011, 2015 | Total care | IQVIA |
| Serbia | 2011, 2015 | Total care | Sales records from marketing authorization holders |
| Tajikistan | 2011, 2015 | Total care | Import records  Certification records |
| Turkey | 2011  2015 | Outpatient  Total care | IQVIA  Wholesaler records from pharmaceutical track and trace system |
| Uzbekistan | 2011, 2015 | Total care | Import records  Sales records from local manufacturers |
| Kosovo* | 2011, 2015 | Total care | Import records |

*in accordance with Security Council resolution 1244 (1999)

#commercial data source provides coverage of around 80-85% of hospital and community sales.

**Table 2: Consumption of ‘Watch’ and ‘Reserve’ group of antibiotics classes as a proportion of total consumption of antibacterials (2015)**

|  | | **DDD/1000 inhabitants per day (DID) and % of total antibiotic consumption** | | | | | | | | | | | | | | | |
| --- | --- | --- | --- | --- | --- | --- | --- | --- | --- | --- | --- | --- | --- | --- | --- | --- | --- |
|  |  | Albania | Armenia | Azerbaijan | Bosnia and Herzegovina | Belarus | Georgia | Kazakhstan | Kyrgyzstan | Kosovo* | Republic of Moldova | Montenegro | Russian federation | Serbia | Tajikistan | Turkey | Uzbekistan |
| Watch group^1^ | DID | 6·6 | 2·9 | 2·4 | 4·2 | 6·3 | 12·7 | 5·7 | 3·7 | 7·1 | 4·3 | 12·2 | 6·4 | 10·7 | 8·3 | 12·8 | 2·7 |
|  | % | 36·2 | 25·4 | 29·5 | 22·0 | 32·7 | 49·5 | 30·7 | 17·3 | 30·5 | 30·0 | 35·4 | 39·8 | 28·8 | 31·6 | 30·4 | 25·3 |
| Reserve group^2^ | DID | 0.01 | 0.01 | <0.01 | <0.01 | 0.16 | 0.06 | 0.02 | <0.01 | <0.01 | <0.01 | 0.01 | 0.03 | 0.03 | 0.01 | 0.05 | 0.01 |
|  | % | 0.04 | 0.09 | 0.01 | 0.02 | 0.83 | 0.25 | 0.1 | 0.01 | 0.01 | 0.01 | 0.02 | 0.2 | 0.09 | 0.04 | 0.12 | 0.12 |
| Total^3^ | DID | 18·3 | 11·3 | 8·2 | 19·3 | 19·3 | 25·7 | 18·6 | 21·2 | 23·3 | 14·2 | 34·4 | 16·2 | 37·1 | 26·2 | 40·2 | 10·5 |

^1^Watch group: Quinolones (J01MA, J01MB); 3rd generation cephalosporins (J01DD); macrolides (J01FA); glycopeptides (J01XA & A07AA09); antipseudomonal penicillins with beta-lactamase inhibitor (J01CR03 & J01CR05); carbapenems (J01DH); faropenem (J01DI03)

^2^Reserve group: Aztreonam (J01DF01); Fourth-generation cephalosporins (J01DE); Fifth-generation cephalosporins (J01DI02, J01DI01, J01DI54); Fosfomycin IV (J01XX01, only parenteral); Oxazolidinone (J01XX08, J01XX11); Polymxyins (J01XB, A07AA10, A07AA05); Tigecycline (J01AA12); Daptomycin (J01XX09)

^3^ Total consumption of antibiotics for this calculation includes J01, Vancomycin (A07AA09), Colistin (A07AA10), Polymyxin B (A07AA05), and Metronidazole (P01AB01).

*in accordance with Security Council resolution 1244 (1999)
