# Supplementary figures and images for: Antimicrobial Medicines Consumption in Eastern Europeand Central Asia – An Updated Cross-National Study and Assessment of QuantitativeMetrics for Policy Action
Source: Front Pharmacol. 2019 Mar 5;9:1156. doi: 10.3389/fphar.2018.01156 (PMC6411709; doi:10.3389/fphar.2018.01156)

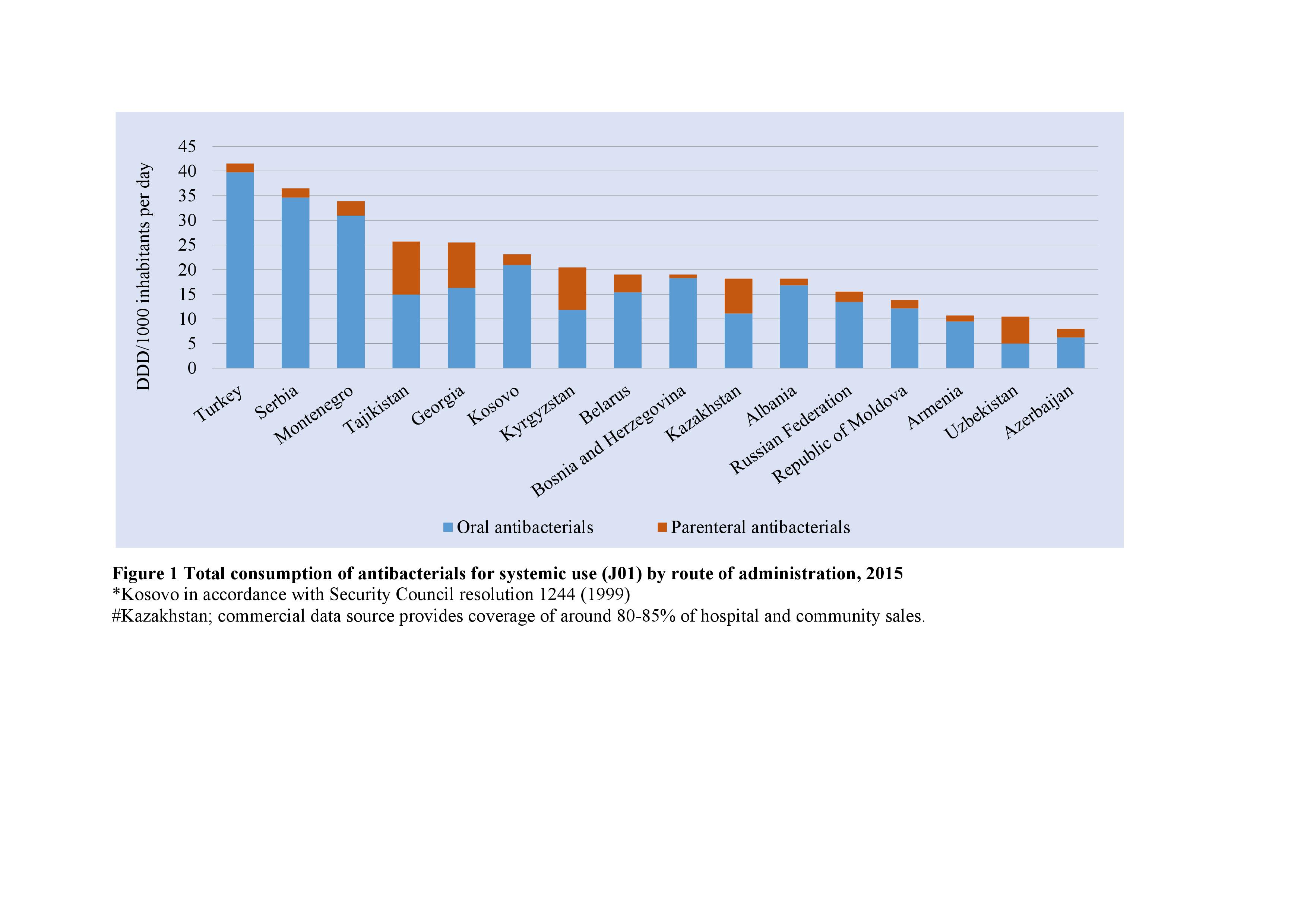

Supplement: Supplementary file 2 [file Image_1.JPEG]
